# Surgical Mortality Risk Scores in Transcatheter Aortic Valve Implantation: Is Their Early Predictive Value Still Strong?

**DOI:** 10.3390/jcdd10060244

**Published:** 2023-05-31

**Authors:** Fortunato Iacovelli, Francesco Loizzi, Alessandro Cafaro, Osvaldo Burattini, Luigi Salemme, Angelo Cioppa, Francesco Rizzo, Chiara Palmitessa, Maurizio D’Alessandro, Daniele De Feo, Armando Pucciarelli, Emanuela De Cillis, Vincenzo Pestrichella, Gaetano Contegiacomo, Tullio Tesorio, Alessandro Santo Bortone

**Affiliations:** 1 Division of University Cardiology, Cardiothoracic Department, Policlinico University Hospital, 70124 Bari, Italy; francescorizzo.lecce.93@gmail.com (F.R.); chiara.palmy@gmail.com (C.P.); maurizio.dalessandro23@gmail.com (M.D.); daniele.df93@gmail.com (D.D.F.); 2 Division of Cardiology, “SS. Annunziata” Hospital, 74121 Taranto, Italy; loizzi91@gmail.com (F.L.); osvaldoburattini@gmail.com (O.B.); 3 Division of Cardiology, “V. Fazzi” Hospital, 73100 Lecce, Italy; dr.alessandrocafaro@libero.it; 4 Interventional Cardiology Service, “Montevergine” Clinic, GVM Care & Research, 83013 Mercogliano, Italy; ginosalemme@hotmail.it (L.S.); cioppa68@gmail.com (A.C.); armandopucciarelli@gmail.com (A.P.); tulliotesorio@gmail.com (T.T.); 5 Division of University Heart Surgery, Cardiothoracic Department, Policlinico University Hospital, 70124 Bari, Italy; emanuela.decillis@gmail.com (E.D.C.); alessandrosanto.bortone@uniba.it (A.S.B.); 6 Interventional Cardiology Service, “Mater Dei” Hospital, 70125 Bari, Italy; vpestrichella@yahoo.it; 7 Interventional Cardiology Service, “Anthea” Clinic, GVM Care & Research, 70124 Bari, Italy; gconteg@gmail.com

**Keywords:** aortic stenosis, transcatheter aortic valve implantation, risk scores, early safety, Valve Academic Research Consortium

## Abstract

Background: Surgical mortality risk scores, even if not properly designed and rarely tested in the transcatheter aortic valve implantation (TAVI) setting, still guide the heart team in managing significant aortic stenosis. Methods: After splitting 1763 consecutive patients retrospectively based on their mortality risk thresholds, the composite endpoint early safety (ES) was adjudicated according to Valve Academic Research Consortium (VARC)-2 and -3 consensus documents. Results: ES incidence was higher if VARC-2 rather than VARC-3 defined. Despite only patients showing VARC-2 ES had significantly lower absolute values of all three main risk scores, these last still failed to foresee both VARC-2 and -3 ES in intermediate-risk patients. The receiver operating characteristic analysis also showed a significant correlation, but with poor diagnostic accuracy, among the three scores and only VARC-2 ES; moreover, the absence of VARC-2 ES and low-osmolar contrast media administration were identified as independent predictors of 1-year mortality and absence of VARC-3 ES, respectively. Finally, even a single complication included in the ES definition could significantly affect 1-year mortality. Conclusion: Currently, the most used mortality risk scores do not have adequate diagnostic accuracy in predicting ES after TAVI. The absence of VARC-2, instead of VARC-3, ES is an independent predictor of 1-year mortality.

## 1. Introduction

Aortic stenosis is the most common primary valve disease in Europe and the U.S.A., whose gold standard treatment has been surgical aortic valve replacement (SAVR) for decades. Despite at its outset, transcatheter aortic valve implantation (TAVI) was considered an attractive, less invasive treatment option only for high surgical risk or selected inoperable patients, randomized controlled trials, such as PARTNER 2 [1], PARTNER 3 [2], the OBSERVANT study [3], NOTION [4], and Evolut Low Risk [5], have recently demonstrated that this percutaneous technique is non-inferior to SAVR in intermediate- and low-surgical-risk patients, too.

The logistic European System for Cardiac Operative Risk Evaluation (EuroSCORE) [6] was the first to be used in clinical practice. Notwithstanding, according to the last European guidelines [7], the calculation of the EuroSCORE II [8,9] and the Society of Thoracic Surgeons Predictive Risk of Mortality (STS-PROM) score [10,11] should guide the heart team in assessing the surgical operative risk and, therefore, in identifying the correct treatment option. Anyhow, while acknowledging that all these surgical risk scores were designed to predict 1-month mortality after surgery, their predictive performance in interventional cardiology has been rarely tested in small groups; in fact, in the TAVI setting, their predictive power is not as highly performing as in patients undergoing SAVR [12,13]. Even if compared to each other, these scores and their threshold values fail to correlate well [14], forcing the heart team to focus on clinical judgment, too.

Valve Academic Research Consortium (VARC) consensus documents [15,16,17] aim to establish clinical assessment and risk stratification of patients suitable for TAVI, and highlight single and composite endpoints. Early safety (ES) is one of the short-term composite endpoints of the VARC-2 consensus document, combining all-cause mortality, all stroke, life-threatening bleeding, stage 2 or 3 acute kidney injury (AKI), coronary artery obstruction requiring intervention, and valve-related dysfunction requiring another aortic valvular procedure within 30 days after TAVI [16]. In the VARC-3 consensus document, the ES definition also includes other adverse events that significantly impact short- and long-term prognoses [17], such as cardiac structural complications, significant aortic regurgitation, and new permanent pacemaker (PM) implantation. The real incidence of ES defined with VARC-2 criteria is 88.3% [18], but nothing is known about the incidence of ES defined according to VARC-3 criteria.

To date, the currently available mortality risk scores have been related to 1-month mortality only in small groups of TAVI patients, so our study aims to evaluate for the first time, in a large population, if these surgical scores could realistically predict ES, defined with both VARC-2 and VARC-3 criteria.

## 2. Materials and Methods

### 2.1. Study Population

This multicenter observational study assessed all consecutive patients who underwent TAVI at five Italian heart centers (Policlinico University Hospital, “Anthea” Clinic and “Mater Dei” Hospital of Bari, “V. Fazzi” Hospital of Lecce, and “Montevergine” Clinic of Mercogliano) involved in the “Magna Graecia” TAVI registry.

Between March 2011 and September 2021, 1763 consecutive patients (982 females, mean age 80.94 ± 5.73, 1667 transfemoral access) suitable for TAVI were enrolled. They underwent preprocedural assessment with transthoracic echocardiography; coronary angiography; computed tomography scan of the heart, aorta, and peripheral arteries; pulmonary function testing (if necessary); carotid artery ultrasonography; and multidisciplinary evaluation by the Valve Team. Most procedures were performed in a standard cardiac catheterization laboratory with the support of anesthesiology and surgical backup by experienced operators. Iodixanol was the only iodinated iso-osmolar CM administered; the other low-osmolar contrast media (LOCM) used for the procedure were iopromide, iobitridol, iohexol, and iomeprol. The deployed valves were balloon-expandable (Edwards Sapien XT and Sapien 3; Meril Myval), self-expanding (Medtronic CoreValve, Engager, Evolut R, and Evolut PRO; Boston Acurate and Acurate neo; Abbott Portico; JenaValve), and others (Boston Lotus; Direct Flow Medical).

Each participating site maintains a prospective database of all TAVI patients treated at that center using the same dedicated archiving software. All baseline demographics; clinical, laboratory, echocardiographic, intraprocedural, and postprocedural data; and hospital outcomes were collected from each patient’s health record; pre-TAVI logistic EuroSCORE, EuroSCORE II, and STS-PROM score were prospectively calculated online using the official websites and calculators based on previously published data, whereas the analysis was performed retrospectively.

Patients’ population was retrospectively split according to the most widespread mortality risk thresholds [7]: low if logistic EuroSCORE, EuroSCORE II and STS-PROM were <10%, <4%, and <4%, respectively; intermediate if they were 10–20%, 4–8%, and 4–8%, respectively; and high if they were ≥20%, ≥8%, and ≥8%, respectively. All the adverse events and the ES composite endpoint were also re-adjudicated retrospectively, by an external committee of interventional cardiologists, according to both VARC-2 and VARC-3 criteria [16,17]. The clinical outcomes analyzed and compared included observed and predicted absence of ES; this last was calculated using the cumulative score percentage of each risk model divided by the number of subjects in the study.

### 2.2. Statistical Analysis

Statistical analysis was performed using SigmaStat 3.5, SPSS 25.0, and STATA 13.0 software. Continuous variables were expressed as the mean ± standard deviation and median (interquartile ranges) of absolute numbers; categorical variables were expressed as frequencies and percentages. As appropriate, comparisons were made by t-test, Mann–Whitney’s U-test, Fisher’s exact test, or χ^2^ test. The normal distribution was assessed with Kolmogorov–Smirnov tests. A receiver operating characteristic (ROC) curve analysis was performed to establish the threshold levels of the mortality risk scores that provided the best cutoff for the absence of ES according to VARC-2 and VARC-3 definitions, but also to establish the minimum number of complications needed to cause 1-year mortality after TAVI. Area under the curve (AUC) values were calculated with confidence intervals (CIs) through concordance statistics to measure test accuracy. The DeLong test was used to identify AUC standard errors. The calibrations of these mortality risk models were evaluated by comparing the mean predicted probability and the mean observed frequency of absence of ES with goodness-of-fit R-squared and Cochran–Armitage tests, calibration plots, and estimation of a calibration slope. After this, new optimal cutoff points for the absence of ES were selected using Youden’s tests, reporting Youden’s indexes; we evaluated sensitivity and specificity according to these new cutoff points. All AUC of mortality risk scores were then compared using the ROC-regression test. Finally, the absence of ES and 1-year mortality predictors was tested in a univariable logistic regression model; all variables with a *p*-value < 0.05 at univariable regression were tested for multicollinearity in a stepwise multivariable model. Only variables with a variance inflation factor <4 were incorporated in the multivariable logistic regression model. The odds ratios (ORs) with 95% CIs were estimated. All statistical tests were two-sided. For all tests, a *p*-value < 0.05 was considered statistically significant.

## 3. Results

### 3.1. Patient Characteristics

A total of 1515 (85.9%) and 1050 (59.6%) patients presented VARC-2- and VARC-3-defined ES, respectively; Supplementary Table S1 and Supplementary Figure S1 describe the distribution of the several component adverse events if the absence of ES was adjudicated with VARC-2 or VARC-3 criteria. All clinical, preprocedural, procedural, and postprocedural data of the study population are shown in Table 1 and Supplementary Table S2.

Body surface area (BSA) and peripheral artery disease (PAD) had a significant impact on the absence of VARC-2 ES (*p* = 0.018 and *p* = 0.008, respectively), whereas the absence of VARC-3 ES occurred more frequently in older (*p* = 0.03) patients already implanted with a permanent PM (*p* = 0.05); no significant differences in sex were observed among ES and no-ES patients (*p* = 0.310 for VARC-2 ES, *p* = 0.872 for VARC-3 ES). Absence of both VARC-2 and VARC-3 ES was detected more in patients with lower baseline values of creatinine clearance (*p* = 0.019 and *p* = 0.015, respectively) and a higher rate of critical preoperative state (*p* = 0.003 and *p* = 0.002, respectively). ES and no-ES patients did not differ significantly with respect to other baseline clinical characteristics.

Moreover, some procedural details, such as the postdilation (*p* < 0.001, for both VARC-2 and VARC-3 ES) of a bioprosthesis other than balloon-expandable (*p* = 0.044 for VARC-2 ES, *p* < 0.001 for VARC-3 ES), the fluoroscopy time (*p* < 0.001 for VARC-2 ES, *p* = 0.018 for VARC-3 ES), the radiation dose (*p* = 0.007 for VARC-2 ES, *p* = 0.037 for VARC-3 ES), and the contrast medium (CM) amount (*p* < 0.001, for both VARC-2 and VARC-3 ES), were all significantly associated with the absence of ES. VARC-2 ES occurred less frequently in the case of valve-in-valve implantation (*p* = 0.014), whereas no VARC-3 ES patients were more likely to receive a small-sized (*p* < 0.001) self-expanding bioprosthesis (*p* < 0.001), administering LOCM (*p* < 0.001).

Concerning complications and outcomes, patients without both VARC-2 and VARC-3 ES exhibited a higher rate of new-onset atrial fibrillation (AF) (*p* < 0.001 for VARC-2 ES, *p* = 0.003 for VARC-3 ES) and mostly needed permanent PM implantation (*p* = 0.016 for VARC-2 ES, *p* < 0.001 for VARC-3 ES) after TAVI. Besides a shorter hospital stay observed in ES patients (5.27 vs. 8.48 days for VARC-2 ES, 4.67 vs. 7.17 days for VARC-3 ES, *p* < 0.001 for both), the occurrence of such early composite endpoints was associated with lower 1-year mortality, too (*p* = 0.002 for VARC-2 ES, *p* = 0.025 for VARC-3 ES).

### 3.2. Surgical Mortality Risk Scores

**Figure 1:**
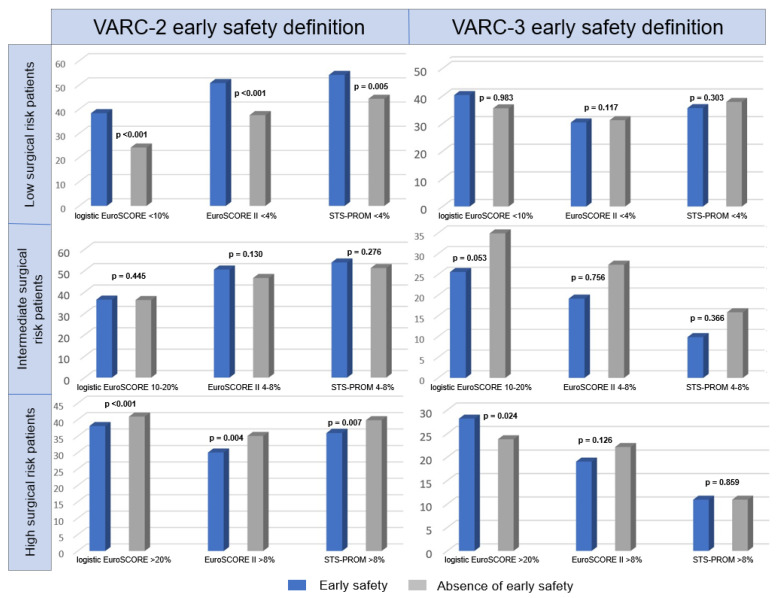
Early safety incidence according to baseline surgical risk profile. VARC = Valve Academic Research Consortium; EuroSCORE = European System for Cardiac Operative Risk Evaluation; STS-PROM = Society of Thoracic Surgery Predictive Risk Of Mortality.

Table 1 also describes the relation between the three surgical mortality risk scores and the absence of ES. As absolute values, these scores were significantly lower (*p* < 0.001) in the VARC-2 ES group; in fact, we found that a significant proportion of patients at higher surgical mortality risk did not develop VARC-2 ES compared with those at lower risk. On the contrary, no statistical difference was detected between patients with or without VARC-3 ES in terms of absolute values of the logistic EuroSCORE (*p* = 0.334), EuroSCORE II (*p* = 0.097), and STS-PROM (*p* = 0.177). Notwithstanding, after splitting the population according to its operative risk profile, the aforementioned significant correlation between the 3 scores and the absence of VARC-2 ES was maintained only in low- and high-risk patients rather than in intermediate-risk patients (*p* = 0.445 if logistic EuroSCORE 10–20%, *p* = 0.130 if EuroSCORE II 4–8%, and *p* = 0.276 if STS-PROM score 4–8%) (Figure 1).

**Figure 2:**
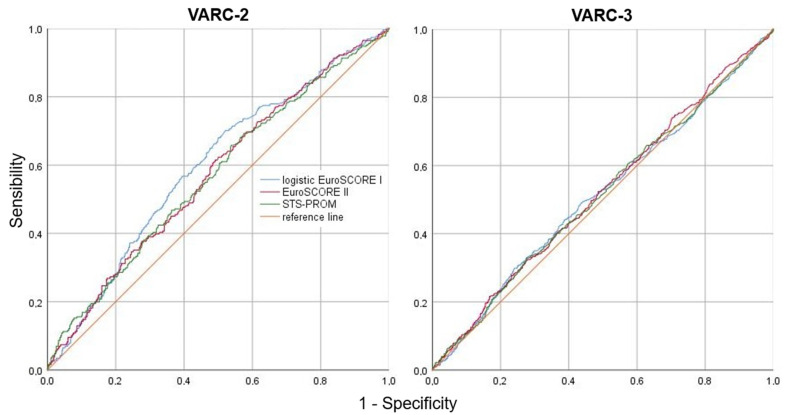
Absence of early safety: ROC curve analysis for surgical mortality risk scores predictive power in TAVI patients. ROC = receiver operating characteristic; EuroSCORE = European System for Cardiac Operative Risk Evaluation; STS-PROM = Society of Thoracic Surgery Predictive Risk Of Mortality.

Moreover, the ratio of observed-to-expected absence of VARC-2 ES (with expected risk calculated on the basis of logistic EuroSCORE, EuroSCORE II, and STS-PROM models) was high for all scores, being 0.890, 0.918, and 0.953, respectively; these scores also correctly estimated the absence of VARC-3 ES with observed-to-expected ratios of 0.992, 0.996, and 0.994, respectively. The ROC analysis also showed a significant correlation between these scores and the absence of ES, only if defined according to the VARC-2 consensus document [15] (Figure 2), but without a significant difference among them (*p* = 0.194).

Nevertheless, based on the AUC of the new cutoff values established with the higher Youden’s indexes, none of the risk scores was significantly performant in detecting the absence of both VARC-2 (logistic EuroSCORE: AUC 0.595, 95% CI 0.571–0.618, sensitivity 50%, specificity 70%, *p* < 0.001; EuroSCORE II: AUC 0.578, 95% CI 0.554–0.601, sensitivity 63%, specificity 51%, *p* < 0.001; STS-PROM: AUC 0.575, 95% CI 0.552–0.558, sensitivity 66%, specificity 46%, *p* < 0.001) and VARC-3 ES (logistic EuroSCORE: AUC 0.514, 95% CI 0.490–0.538, sensitivity 30%, specificity 76%, *p* = 0.334; EuroSCORE II: AUC 0.523, 95% CI 0.499–0.547, sensitivity 53%, specificity 51%, *p* = 0.097; STS-PROM: AUC 0.519, 95% CI 0.495–0.543, sensitivity 33%, specificity 75%, *p* = 0.177).

In the end, ROC analysis was also performed to evaluate the correlation between the number of complications included in the composite endpoint ES and 1-year mortality: depending on the newly established cutoff value (1 ± 0.062), even a single complication could significantly affect 1-year mortality, with sufficient predictive performance (AUC 0.646, 95% CI 0.624–0.669, sensitivity 53%, specificity 86%, *p* < 0.001) (Table 2).

### 3.3. Absence of Early Safety and 1-Year Mortality Predictors

Univariable and multivariable analysis models were built using logistic regression (Table 3); various parameters, i.e., BSA, PAD, pulmonary arterial systolic pressure, surgical risk scores, prior SAVR, postdilation, procedural and fluoroscopy times, radiation dose, CM volume, and new-onset AF post-TAVI, were found to be significantly associated with the absence of VARC-2 ES. Conversely, type of valve, postdilation, fluoroscopy time, radiation dose, LOCM administration, CM volume, and new-onset AF were found to be significantly associated with the absence of VARC-3 ES. Nevertheless, only postdilation (OR 1.962; 95% CI 1.042–3.695; *p* = 0.037) and new-onset AF (OR 3.220; 95% CI 1.536–6.750; *p* = 0.002) have been identified as independent predictors of the absence of VARC-2 ES, whereas administration of LOCM (OR 2.876; 95% CI 1.582–5.228, *p* = 0.001) was identified as the only independent predictor of the absence of VARC-3 ES.

Moreover, in univariable analyses, 10 baseline/procedural risk factors and outcomes were also found to be significantly associated with 1-year mortality. At multivariable analysis, the absence of only VARC-2 ES was found to be an independent predictor of 1-year mortality (OR 2.263; 95% CI 1.021–5.018, *p* = 0.044); other independent predictors were EuroSCORE II (OR 1.060; 95% CI 1.013–1.108, *p* = 0.011), self-expanding prosthesis implantation (OR 0.270; 95% CI 0.143–0.509, *p* < 0.001), and new-onset AF post TAVI (OR 2.558; 95% CI 1.213–5.392; *p* = 0.014).

## 4. Discussion

Despite early and late outcomes also depending on several TAVI-specific factors, including patient frailty, experience of the heart team, prosthesis type, procedural volume, and learning curve, surgical mortality risk scores are still the most used to predict them. The main findings of our study were (1) the accuracy of the commonly used risk scores in predicting TAVI-related ES is not as efficient as expected; (2) after splitting the population according to risk scores’ thresholds, VARC-2 and VARC-3 ES cannot be foreseen for the intermediate-risk patients; (3) absence of VARC-2, instead of VARC-3 ES, is an independent predictor of 1-year mortality; (4) each adverse event included in the composite endpoint ES, by itself, can have a significant influence, without an additive effect, on 1-year mortality.

### 4.1. Early Safety

After the new definition of ES in the last updated consensus document [16], ours is the largest TAVI cohort in which this early composite endpoint, defined with both VARC-2 and VARC-3 criteria, has been analyzed. Our incidence of ES, if VARC-2 defined, is in line with data reported in the literature [19], and its absence was mostly related to life-threatening bleedings and major vascular complications. Conversely, type 2–4 bleedings; major vascular, access-related, or cardiac structural complications; moderate-to-severe aortic regurgitation; and permanent PM implantation were the most frequently responsible for the absence of VARC-3 ES: the lower incidence of VARC-3 ES with respect to VARC-2 ES could be properly interpreted as being the last definition of this composite endpoint much more inclusive of complications.

To date, other studies have used the VARC-3 consensus document to adjudicate events: our data confirm what Costa et al. [20] have already highlighted in a small cohort of patients about the higher rate of the composite endpoint ES after balloon-expandable prostheses implantation. Moreover, another important known contributor to reducing VARC-2 ES occurrence is the administration of large amounts of LOCM: in fact, in line with what was previously stated in the literature [21,22], low instead of iso-osmolality of CM seems to have an unfavorable effect on renal function, raising acute kidney injury incidence. Notwithstanding, only osmolality, rather than CM volume, seems to be an independent VARC-3 ES predictor in our TAVI population.

### 4.2. Surgical Mortality Risk Scores and Absence of Early Safety

Consistent with its main aim, this study provides for the first time an original use of currently adopted surgical mortality risk scores to predict the absence of ES after TAVI, as these scores have only been tested so far to predict 1-month and 1-year mortality in this setting, with limited diagnostic accuracy [13]. Although only the STS-PROM score seemed to foresee 1-month mortality [22], some authors have already demonstrated its lack of discriminatory power and no difference in prediction accuracy compared to other scores [23,24]. In our cohort, all the risk scores did not demonstrate significant differences between the predicted and observed absence of VARC-2 and VARC-3 ES. However, although all patients who failed to achieve VARC-2 ES had significantly higher absolute values of the three risk scores, these last showed limited diagnostic accuracy, as their AUC was never above 0.6; if ES was adjudicated according to the VARC-3 definition, the diagnostic accuracy of the 3 mortality risk scores was even lower, as their AUC was never above 0.5. All these findings are almost certainly because such risk scores have been developed and validated in different settings. In fact, they showed adequate diagnostic accuracy in predicting operative mortality after isolated SAVR [25].

Finally, no study has analyzed the predictive performance of such risk scores based on the preprocedural risk category, i.e., low, intermediate, or high. Instead, our low- and high-risk patients showed a significant association between their surgical risk scores and the absence of VARC-2 ES. In contrast, no significant correlation was found in any preprocedural risk category if the absence of ES was VARC-3-defined.

### 4.3. One-Year Mortality Predictors

An attempt to identify predictors of 1-year mortality after TAVI was made, too. Despite the findings of the small study by Compagnone et al., the STS-PROM score was the only score able to stratify long-term all-cause and cardiovascular mortality, and EuroSCORE II was the only risk score significantly related to the 1-year mortality of our population. Nevertheless, we agree with other studies about the lower performance of these scores in predicting long-term events in the post-TAVI rather than post-SAVR setting [13,26], leading to the urgent need for a specific TAVI-tailored risk score [24].

According to our analysis, other procedural or postprocedural features can have a negative impact on 1-year mortality, first of all, and in line with the literature [27], self-expanding technologies, probably due to their deployment characteristics. In addition, new-onset AF has been identified as another independent predictor of 1-year mortality, too. In fact, AF is independently associated with an almost twofold increased risk of all-cause mortality [28]; such a novel finding could be likely related to the higher frailty of AF patients, their anticoagulation need, and their higher risk of thromboembolic events.

Finally, even the absence of VARC-2 ES has been shown to be an independent predictor of 1-year mortality. This information can properly be explained by the possible limits of new VARC-3 criteria: the more complications are included in this composite endpoint, the more its long-term predictive performance will decrease. In fact, the newsworthy twist of our analysis is that adverse events do not have an additive effect in influencing 1-year mortality: a single adverse event is enough to affect 1-year mortality.

### 4.4. Study Limitations

Although it was obtained from a prospectively collected database, this is an unspecified post hoc analysis. Therefore, we cannot exclude that potential confounding factors not considered in the model may have influenced the results. The effect of a learning curve and changes in treatment strategy is also heterogeneous, as the study spanned more than a decade, during which several updates to the STS-PROM score have also been conducted. Furthermore, we believe that aspects of management not controlled or specified may have been a source of bias. Finally, an independent committee did not adjudicate all clinical events that were site-referred.

## 5. Conclusions

Unfortunately, available surgical risk scores have demonstrated limits in predicting both short- and long-term mortality and the absence of ES after TAVI, especially in intermediate-risk patients, who account for the largest proportion of patients currently undergoing this transcatheter procedure. Defining the composite endpoint ES according to the most recent VARC-3 consensus document significantly reduced its incidence, probably due to the inclusion of additional frequent adverse events, which have also been shown to influence 1-year mortality.

Moreover, EuroSCORE II and the absence of VARC-2, instead of the absence of VARC-3 ES, are independent predictors of 1-year mortality, albeit LOCM administration is the only independent predictor of the absence of VARC-3 ES, confirming the paramount importance of choosing an iso-osmolar contrast medium also in this setting of patients.

Nevertheless, until a better performing TAVI-dedicated risk score is created, individual clinical judgment in a heart team approach should be considered the fundamental weapon to stratify TAVI patients in a precise and detailed manner.

## Figures and Tables

**Table 1:** Baseline characteristics, procedural features, and outcomes of the study population according to early safety incidence (n = 1763).

Variable	All	VARC-2 Early Safety		*p*	VARC-3 Early Safety		*p*
		Yes (n = 1515)	No (n = 248)		Yes (n = 1050)	No (n = 713)	
***Patient characteristics***							
Age (years)	80.94 ± 5.73	80.95 ± 5.63	80.90 ± 6.34	0.361	80.76 ± 5.62	81.21 ± 5.90	0.030
Female	982 (55.70%)	836 (55.18%)	146 (58.87%)	0.310	587 (55.90%)	395 (55.40%)	0.872
Body Surface Area (m^2^)	1.75 ± 0.17	1.76 ± 0.17	1.73 ± 0.19	0.018	1.76 ± 0.17	1.75 ± 0.17	0.100
***Mortality risk scores***							
Logistic EuroSCORE (%)	16.21 ± 12.41	15.78 ± 12.23	18.92 ± 13.23	<0.001	16.02 ± 12.40	16.51 ± 12.43	0.334
<10	617/1695 (36.40%)	561/1463 (38.35%)	56/232 (24.14%)	<0.001	376/1031 (36.47%)	241/664 (36.29%)	0.983
10–20	652/1695 (38.47%)	557/1463 (38.07%)	95/232 (40.95%)	0.445	416/1031 (40.35%)	236/664 (35.54%)	0.053
≥20	426/1695 (25.13%)	345/1463 (23.58%)	81/232 (34.91%)	<0.001	239/1031 (23.18%)	187/664 (28.16%)	0.024
EuroSCORE II (%)	5.91 ± 5.83	5.72 ± 5.59	7.05 ± 7.02	<0.001	5.70 ± 5.39	6.21 ± 6.42	0.097
<4	856/1749 (48.94%)	764/1504 (50.80%)	92/245 (37.55%)	<0.001	528/1045 (50.53%)	328/704 (46.59%)	0.117
4–8	538/1749 (30.76%)	452/1504 (30.05%)	86/245 (35.10%)	0.130	318/1045 (30.43%)	220/704 (31.25%)	0.756
≥8	355/1749 (20.30%)	288/1504 (19.15%)	67/245 (27.35%)	0.004	199/1045 (19.04%)	156/704 (22.16%)	0.126
STS-PROM (%)	4.73 ± 3.56	4.57 ± 3.10	5.71 ± 5.49	<0.001	4.60 ± 3.07	4.93 ± 4.16	0.177
<4	923/1749 (52.78%)	814/1503 (54.16%)	109/246 (44.31%)	0.005	562/1044 (53.83%)	361/705 (51.21%)	0.303
4–8	639/1749 (36.53%)	541/1503 (35.99%)	98/246 (39.84%)	0.276	372/1044 (35.63%)	267/705 (37.87%)	0.366
≥8	187/1749 (10.69%)	148/1503 (9.85%)	39/246 (15.85%)	0.007	110/1044 (10.54%)	77/705 (10.92%)	0.859
***Procedural details***							
Valve-in-valve	79 (4.48%)	60 (3.96%)	19 (7.66%)	0.014	48 (4.57%)	31 (4.35%)	0.916
Predilation	828/1751 (47.29%)	712/1505 (47.31%)	116/246 (47.15%)	0.981	475/1043 (45.54%)	353/708 (49.86%)	0.084
Valve substitute							
Balloon-expandable	578 (32.79%)	511 (33.73%)	67 (27.02%)	0.044	396 (37.71%)	182 (25.53%)	<0.001
Self-expanding	1097 (62.22%)	935 (61.72%)	162 (65.32%)	0.310	601 (57.24%)	496 (69.56%)	<0.001
Others	88 (4.99%)	69 (4.55%)	19 (7.66%)	0.054	53 (5.05%)	35 (4.91%)	0.984
Valve size ≤ 26 mm	1079 (61.20%)	934 (61.65%)	145 (58.47%)	0.377	685 (65.24%)	394 (55.26%)	<0.001
Postdilation	449/1755 (25.58%)	361/1508 (23.94%)	88/247 (35.63%)	<0.001	222/1047 (21.20%)	227/708 (32.06%)	<0.001
Fluoroscopy time (min)	23.94 ± 15.97	22.60 ± 12.72	32.61 ± 27.86	<0.001	22.42 ± 13.03	26.42 ± 19.61	0.018
Radiation dose (mGy)	1151.20 ± 866.71	1103.30 ± 756.78	1490.40 ± 1378.69	0.007	1099.49 ± 778.35	1237.11 ± 991.67	0.037
Procedural time (min)	93.35 ± 31.54	91.13 ± 29.51	104.79 ± 38.95	0.026	91.31 ± 28.88	95.31 ± 33.93	0.398
LOCM	1312/1747 (75.10%)	1124/1503 (74.78%)	188/244 (77.05%)	0.497	751/1044 (71.93%)	561/703 (79.80%)	<0.001
CM volume (mL)	149.85 ± 72.43	145.15 ± 65.77	178.82 ± 99.93	<0.001	144.17 ± 65.92	158.25 ± 80.44	<0.001
***Other complications and outcomes***							
Permanent pacemaker implantation	210/1538 (13.65%)	170/1330 (12.78%)	40/208 (19.23%)	0.016	0/907 (0.00%)	210/631 (33.28%)	<0.001
New-onset LBBB	518 (29.38%)	445 (29.37%)	73 (29.43%)	0.956	311 (29.62%)	207 (29.03%)	0.832
New-onset atrial fibrillation	140/1425 (9.82%)	105/1235 (8.50%)	35/190 (18.42%)	<0.001	67/855 (7.84%)	73/570 (12.81%)	0.003
Post-TAVI hospital length of stay (days)	5.64 ± 4.21	5.27 ± 3.77	8.48 ± 5.94	<0.001	4.67 ± 2.63	7.17 ± 5.78	<0.001
1-year mortality	64 (3.63%)	46 (3.04%)	18 (7.26%)	0.002	29 (2.76%)	35 (4.91%)	0.025

VARC = Valve Academic Research Consortium; EuroSCORE = European System for Cardiac Operative Risk Evaluation; STS-PROM = Society of Thoracic Surgery Predictive Risk Of Mortality; LOCM = low-osmolar contrast medium; LBBB = left bundle branch block; TAVI = transcatheter aortic valve implantation.

**Table 2:** ROC analysis for the prediction of absence of early safety by dedicated scores, and 1-year mortality by the number of complications.

	AUC (DeLong Standard Error)	95% CI	Asymptotic Significance	CL	Slope	Cutoff	Youden Index	Sensitivity (%)	Specificity (%)
***Absence of early safety (VARC-2)***									
Logistic EuroSCORE (%)	0.595 ± 0.020	0.571–0.618	<0.001	0.00	1.000	11.36 ± 0.03	0.177	70	48
EuroSCORE II (%)	0.578 ± 0.019	0.554–0.601	<0.001	−0.00	1.000	3.97 ± 0.03	0.139	63	51
STS-PROM (%)	0.575 ± 0.020	0.552–0.598	<0.001	0.00	1.000	3.53 ± 0.03	0.125	66	46
***Absence of early safety (VARC-3)***									
Logistic EuroSCORE (%)	0.514 ± 0.014	0.490–0.538	0.334	−0.00	1.000	19.41 ± 0.02	0.057	30	76
EuroSCORE II (%)	0.523 ± 0.014	0.499–0.547	0.097	0.00	1.000	4.02 ± 0.02	0.046	53	51
STS-PROM (%)	0.519 ± 0.014	0.495–0.543	0.177	0.00	1.000	5.21 ± 0.02	0.058	33	75
***1-year mortality***									
Number of complications	0.646 ± 0.040	0.624–0.669	<0.001	−0.00	1.000	1 ± 0.062	0.395	53	86

ROC = receiver operating characteristic; AUC = area under the curve; CI = confidence interval; CL = calibration in the large; VARC = Valve Academic Research Consortium; EuroSCORE = European System for Cardiac Operative Risk Evaluation; STS-PROM = Society of Thoracic Surgery Predictive Risk Of Mortality.

**Table 3:** Absence of early safety and 1-year mortality predictors.

Predictors	Absence of Early Safety (VARC-2)				Absence of Early Safety (VARC-3)				1-Year Mortality			
	Univariable OR (95% CI)	*p*	Multivariable OR (95% CI)	*p*	Univariable OR (95% CI)	*p*	Multivariable OR (95% CI)	*p*	Univariable OR (95% CI)	*p*	Multivariable OR (95% CI)	*p*
Body Surface Area	0.333 (0.150–0.738)	0.007			0.626 (0.359–1.094)	0.100						
PAD	1.499 (1.122–2.002)	0.006	1.477 (0.749–2.915)	0.260	1.094 (0.882–1.358)	0.415						
Pulmonary arterial systolic pressure	1.014 (1.004–1.024)	0.006			1.003 (0.996–1.011)	0.402						
Logistic EuroSCORE	1.018 (1.008–1.027)	<0.001			1.003 (0.995–1.011)	0.433			1.028 (1.013–1.043)	<0.001		
EuroSCORE II	1.032 (1.012–1.052)	<0.001	1.012 (0.946–1.082)	0.734	1.015 (0.999–1.032)	0.074			1.066 (1.039–1.093)	<0.001	1.060 (1.013–1.108)	0.011
STS-PROM	1.072 (1.038–1.108)	<0.001	1.077 (0.958–1.212)	0.214	1.026 (0.999–1.054)	0.062			1.087 (1.041–1.134)	<0.001	1.055 (0.969–1.148)	0.217
Valve-in-valve	2.012 (1.179–3.433)	0.010	0.356 (0.043–2.948)	0.338	0.949 (0.598–1.506)	0.824						
Self-expanding prosthesis	1.169 (0.882–1.548)	0.278			1.708 (1.397–2.088)	<0.001	0.978 (0.615–1.555)	0.925	0.225 (0.129–0.3919	<0.001	0.270 (0.143–0.509)	<0.001
Postdilation	1.758 (1.321–2.341)	<0.001	1.962 (1.042–3.695)	0.037	1.754 (1.412–2.178)	<0.001	1.284 (0.803–2.052)	0.296				
Fluoroscopy time (min)	1.031 (1.020–1.042)	<0.001	1.014 (0.995–1.033)	0.160	1.017 (1.008–1.026)	<0.001	1.010 (0.994–1.026)	0.212				
Radiation dose (mGy)	1.000 (1.000–1.001)	<0.001	1.000 (0.999–1.000)	0.121	1.000 (1.000–1.000)	0.025	1.000 (0.999–1.000)	0.877				
Procedural time (min)	1.013 (1.002–1.023)	0.016			1.004 (0.996–1.012)	0.333						
LOCM	1.132 (0.822–1.559)	0.448			1.541 (1.227–1.937)	<0.001	2.876 (1.582–5.228)	0.001				
CM volume	1.005 (1.004–1.007)	<0.001			1.003 (1.001–1.004)	<0.001			1.004 (1.001–1.006)	0.013	1.002 (0.999–1.006)	0.230
Number of complications									1.956 (1.578–2.425)	<0.001		
New-onset LBBB									0.434 (0.219–0.852)	0.017	0.513 (0.242–1.088)	0.082
New-onset atrial fibrillation	2.430 (1.600–3.690)	<0.001	3.220 (1.536–6.750)	0.002	1.727 (1.217–2.452)	0.002	1.338 (0.728–2.457)	0.348	2.654 (1.329–5.299)	0.006	2.558 (1.213–5.392)	0.014
Absence of early safety (VARC-2)									2.499 (1.424–4.386)	0.001	2.263 (1.021–5.018)	0.044
Absence of early safety (VARC-3)									1.817 (1.101–3.001)	0.020	1.638 (0.793–3.383)	0.182

VARC = Valve Academic Research Consortium; OR = odds ratio; CI = confidence interval; PAD = peripheral arterial disease; EuroSCORE = European System for Cardiac Operative Risk Evaluation; STS-PROM = Society of Thoracic Surgery Predictive Risk Of Mortality; LOCM = low-osmolar contrast medium; LBBB = left bundle branch block.

## Data Availability

Data are accessible with the article (raw data are available upon individual request).

## Supplementary Materials

The following supporting information can be downloaded at: https://www.mdpi.com/article/10.3390/jcdd10060244/s1, Figure S1: (a) Absence of VARC-2 ES: distribution of the component adverse events in the study population, (b) Absence of VARC-3 ES: distribution of the component adverse events in the study population; Table S1: (a) Absence of VARC-2 ES: distribution of the component adverse events in the study population (n = 248), (b) Absence of VARC-3 ES: distribution of the component adverse events in the study population (n = 713); Table S2: Other baseline characteristics and procedural features of the study population according to early safety incidence (n = 1763).
