# Supplementary material for: Surgical Mortality Risk Scores in Transcatheter Aortic Valve Implantation: Is Their Early Predictive Value Still Strong?
Source: J Cardiovasc Dev Dis. 2023 May 31;10(6):244. doi: 10.3390/jcdd10060244 (PMC10298866; doi:10.3390/jcdd10060244)
Supplement: Supplementary file 1 [file jcdd-10-00244-s001.zip › SupplementaryTable1.pdf]

Supplementary Table 1a. Absence of VARC-2 early safety: distribution of the component adverse events in the study population (n=248).

Variable	Intra/periprocedural mortality		Stroke		Life-threatening bleeding		Stage 2-3 AKI		Coronary artery obstruction requiring intervention		Major vascular complication		Valve-related dysfunction requiring repeat procedure	
	Yes	No	Yes	No	Yes	No	Yes	No	Yes	No	Yes	No	Yes	No
Intra/periprocedural mortality (n=45)			2	43	10	35	6	39	5	40	8	37	13	32
Stroke (n=30)	2	28			0	30	1	29	2	28	7	23	3	27
Life-threatening bleeding (n=50)	10	40	1	49			11	39	2	48	34	16	19	31
Stage 2-3 AKI (n=58)	6	52	0	58	11	47			0	58	17	41	8	50
Coronary artery obstruction requiring intervention (n=13)	5	8	1	12	2	11	0	13			1	12	10	3
Major vascular complication (n=122)	8	114	6	116	16	110	17	105	1	121			18	104
Valve-related dysfunction requiring repeat procedure (n=15)	2	13	0	15	4	11	2	13	1	14	5	10		

VARC = Valve Academic Research Consortium; AKI = acute kidney injury.

Supplementary Table 1b. Absence of VARC-3 early safety: distribution of the component adverse events in the study population (n=713).

Variable	Intra/periprocedural mortality		Stroke		Type 2-4 bleeding		Major vascular, access-related, or cardiac structural complication		Stage 3-4 AKI		Moderate-to-severe aortic regurgitation		Permanent PM implantation		Surgery or intervention related to the device	
	Yes	No	Yes	No	Yes	No	Yes	No	Yes	No	Yes	No	Yes	No	Yes	No
Intra/periprocedural mortality (n=45)			2	43	15	30	20	25	5	40	3	42	4	21*	4	41
Stroke (n=30)	2	28			12	180	5	25	0	30	1	29	7	22*	1	29
Type 2-4 bleeding (n=415)	15	400	13	402			142	273	21	394	45	370	59	303*	35	380
Major vascular, access-related, or cardiac structural complication (n=168)	20	148	7	161	26	142			13	155	23	145	25	114*	24	144
Stage 3-4 AKI (n=34)	5	29	0	34	13	21	21	13			2	32	8	20*	3	31
Moderate-to-severe aortic regurgitation (n=124)	3	121	3	121	45	79	23	101	2	122			18	91*	65	59
Permanent PM implantation (n=210)	4	206	8	202	59	151	25	185	8	202	18	192			11	199
Surgery or intervention related to the device (n=15)	2	13	0	15	5	10	6	9	1	14	1	14	2	11*		

VARC = Valve Academic Research Consortium; AKI = acute kidney injury; PM = pacemaker.

[*] All patients already implanted with permanent pacemaker, implantable cardioverter-defibrillator or cardiac resynchronization therapy before transcatheter aortic valve implantation have been considered early safe if free from other single adverse events of such composite endpoint.
