# Supplementary material for: Surgical Mortality Risk Scores in Transcatheter Aortic Valve Implantation: Is Their Early Predictive Value Still Strong?
Source: J Cardiovasc Dev Dis. 2023 May 31;10(6):244. doi: 10.3390/jcdd10060244 (PMC10298866; doi:10.3390/jcdd10060244)
Supplement: Supplementary file 1 [file jcdd-10-00244-s001.zip › SupplementaryTable2.pdf]

Supplementary Table 2. Other baseline characteristics and procedural features of the study population according to early safety incidence (n=1763).

Variable	All	VARC-2 early safety		p value	VARC-3 early safety		p value
		Yes (n=1515)	No (n=248)		Yes (n=1050)	No (n=713)	
Patient characteristics							
Body Mass Index (kg/m^2)	27.41±4.60	27.45±4.56	27.15±4.85	0.276	27.55±4.61	27.20±4.58	0.135
Hypertension	1671/1761 (94.89%)	1436/1513 (94.91%)	235 (94.76%)	0.957	999/1048 (95.32%)	672/713 (94.25%)	0.371
Diabetes mellitus	544/1762 (30.87%)	463/1514 (30.58%)	81 (32.66%)	0.560	331/1049 (31.55%)	213 (29.87%)	0.486
Insulin-dependent	216/1734 (12.46%)	180/1492 (12.06%)	36/242 (14.88%)	0.261	129/1040 (12.40%)	87/694 (12.54%)	0.994
Dyslipidemia	1148/1760 (65.23%)	989/1512 (65.41%)	159 (64.11%)	0.745	689/1047 (65.81%)	459/713 (64.37%)	0.570
Smoking	115 (6.52%)	98 (6.47%)	17 (6.85%)	0.929	66 (6.29%)	49 (6.87%)	0.696
Anemia*	961/1757 (54.69%)	815/1510 (53.97%)	146/247 (59.11%)	0.152	585/1046 (55.93%)	376/711 (52.88%)	0.227
COPD	455/1762 (25.82%)	381/1514 (25.16%)	74/247 (29.84%)	0.139	273/1049 (26.02%)	182 (25.53%)	0.858
Neurological dysfunction	148/1756 (8.43%)	124/1510 (8.21%)	24/246 (9.76%)	0.494	86/1047 (8.21%)	62/709 (8.74%)	0.760
Severe liver disease	37/1760 (2.10%)	32/1512 (2.12%)	5 (2.02%)	0.891	28 (2.67%)	9 (1.26%)	0.064
PAD	460/1757 (26.18%)	378/1511 (25.02%)	82/246 (33.33%)	0.008	267/1048 (25.47%)	193/709 (27.22%)	0.447
Carotid stenosis ≥50%	289 (16.39%)	248 (16.37%)	41 (16.53%)	0.977	175 (16.67%)	114 (15.99%)	0.755
Critical preoperative state	71/1755 (4.04%)	52/1509 (3.45%)	19/246 (7.72%)	0.003	29 (2.77%)	42/710 (5.91%)	0.002
CAD history	424/1760 (24.09%)	365/1513 (24.12%)	59/247 (23.89%)	0.999	255/1048 (24.32%)	169/712 (23.74%)	0.818
Prior myocardial infarction	234/1762 (13.28%)	197/1514 (13.01%)	37 (14.92%)	0.472	140/1049 (13.35%)	94 (13.18%)	0.978
Prior cardiac surgery	261/1761 (14.82%)	223/1513 (14.74%)	38 (15.32%)	0.887	165 (15.71%)	96/711 (13.50%)	0.225
Far from TAVI prior myocardial revascularization	392/1761 (22.26%)	339/1513 (22.41%)	53 (21.37%)	0.779	236/1049 (22.50%)	156/712 (21.91%)	0.816
PCI	234/1761 (13.29%)	202/1513 (13.35%)	32 (12.90%)	0.927	136/1049 (12.96%)	98/712 (13.76%)	0.679

CABG	99/1761 (5.62%)	83/1513 (5.49%)	16 (6.45%)	0.643	63/1049 (6.01%)	36/712 (5.06%)	0.457
PCI + CABG	59/1761 (3.35%)	54/1513 (3.57%)	5 (2.02%)	0.285	37/1049 (3.53%)	22/712 (3.09%)	0.715
Close to TAVI myocardial revascularization†	266/1758 (15.13%)	226/1512 (14.95%)	40/246 (16.26%)	0.662	147/1048 (14.03%)	119/710 (16.76%)	0.133
PCI	259/1758 (14.73%)	220/1512 (14.55%)	39/246 (15.85%)	0.661	143/1048 (13.64%)	116/710 (16.34%)	0.135
CABG	5/1758 (0.28%)	4/1512 (0.26%)	1/246 (0.41%)	0.797	3/1048 (0.28%)	2/712 (0.29%)	0.661
PCI + CABG	2/1758 (0.11%)	2/1512 (0.13%)	0/246 (0.00%)	0.653	1/1048 (0.09%)	1/712 (0.14%)	0.657
Residual significant CAD during TAVI	222/1754 (12.66%)	185/1507 (12.28%)	37/247 (14.98%)	0.280	142/1044 (13.60%)	80/710 (11.27%)	0.171
Prior PM/ICD/CRT implantation	217 (12.31%)	185 (12.21%)	32 (12.90%)	0.839	143 (13.62%)	74 (10.38%)	0.050
NYHA functional class III-IV	1536 (87.12%)	1318 (87.00%)	218 (87.90%)	0.770	919 (87.52%)	617 (86.53%)	0.592
Baseline renal function assessment							
Pre-TAVI CKD‡	750 (42.54%)	633 (41.78%)	117 (47.18%)	0.128	433 (41.24%)	317 (44.46%)	0.196
SCr (mg/dL)	1.15±0.72	1.14±0.73	1.19±0.67	0.397	1.13±0.71	1.17±0.73	0.241
CrCl (mL/min)	54.81±22.52	55.17±21.84	52.74±26.27	0.019	55.72±21.91	53.47±23.34	0.015
eGFR (mL/min/1.73 m^2)	66.65±25.20	66.98±24.94	64.64±26.59	0.164	67.30±24.95	65.70±25.55	0.203
Electrocardiography							
Sinus rhythm	1463 (82.98%)	1264 (83.43%)	199 (80.24%)	0.251	873 (83.14%)	590 (82.75%)	0.880
Paroxysmal atrial fibrillation anamnesis	217 (12.31%)	184 (12.14%)	33 (13.31%)	0.681	123 (11.71%)	94 (13.18%)	0.397
Chronic or persistent atrial fibrillation / flutter	300 (17.02%)	251 (16.57%)	49 (19.76%)	0.251	177 (16.86%)	123 (17.25%)	0.880
PM-induced rhythm	98 (5.55%)	85 (5.61%)	13 (5.24%)	0.932	62 (5.90%)	36 (5.05%)	0.507
Echocardiography							
LVEF (%)	52.94±10.40	53.07±10.32	52.14±10.87	0.193	53.19±10.53	52.59±10.21	0.281
Peak aortic gradient (mmHg)	76.01±21.65	76.03±21.47	75.85±21.47	0.996	76.31±21.62	75.52±21.70	0.635
Mean aortic gradient (mmHg)	46.30±14.72	46.45±14.56	45.42±15.62	0.319	46.54±14.49	45.96±15.04	0.362
Moderate-to-severe aortic regurgitation	379 (21.50%)	324 (21.39%)	55 (22.18%)	0.843	222 (21.14%)	157 (22.02%)	0.703
Moderate-to-severe mitral regurgitation	668/1672 (39.95%)	567/1437 (39.46%)	101/235 (42.98%)	0.342	391/989 (39.53%)	277/683 (40.56%)	0.713

Pulmonary artery systolic pressure (mmHg)	39.43±13.57	39.04±13.09	41.80±15.99	0.070	39.19±12.96	39.80±14.46	0.962
CT-guided procedure	1594 (90.41%)	1372 (90.56%)	222 (89.52%)	0.688	953 (90.76%)	641 (89.90%)	0.603
Procedural details							
Transfemoral access route	1667 (94.55%)	1438 (94.92%)	229 (92.34%)	0.132	995 (94.76%)	672 (94.25%)	0.720
Other access routes	96 (5.45%)	77 (5.08%)	19 (7.66%)	0.132	55 (5.24%)	41 (5.75%)	
transsubclavian	28 (1.59%)	22 (1.45%)	6 (2.42%)	0.392	19 (1.81%)	9 (1.26%)	0.479
transapical	58 (3.29%)	48 (3.17%)	10 (4.03%)	0.607	33 (3.14%)	25 (3.51%)	0.777
direct aortic	8 (0.45%)	5 (0.33%)	3 (1.21%)	0.161	1 (0.09%)	7 (0.98%)	0.018
Orotracheal intubation	243 (13.78%)	202 (13.33%)	41 (16.53%)	0.209	152 (14.48%)	91 (12.76%)	0.340

VARC = Valve Academic Research Consortium; COPD = chronic obstructive pulmonary disease; PAD = peripheral arterial disease; CAD = coronary artery disease; TAVI = transcatheter aortic valve implantation; PCI = percutaneous coronary intervention; CABG = coronary artery by-pass grafting; PM = pacemaker; ICD = implantable cardioverter-defibrillator; CRT = cardiac resynchronization therapy; NYHA = New York Heart Association; CKD = chronic kidney disease; SCr = serum creatinine; CrCl = creatinine clearance; eGFR = estimated glomerular filtration ratio; LVEF = left ventricular ejection fraction; CT = computed tomography.

[*] Preprocedural anemia was defined by the World Health Organization definition of anemia: hemoglobin <12 g/dL for women and <13 g/dL for men.

[†] Percutaneous and/or surgical myocardial revascularization performed no more than 1 month before TAVI.

[‡] CKD was defined as baseline eGFR of < 60 mL/min/1.73 m^2.
