# Supplementary figures and images for: Surgical Mortality Risk Scores in Transcatheter Aortic Valve Implantation: Is Their Early Predictive Value Still Strong?
Source: J Cardiovasc Dev Dis. 2023 May 31;10(6):244. doi: 10.3390/jcdd10060244 (PMC10298866; doi:10.3390/jcdd10060244)

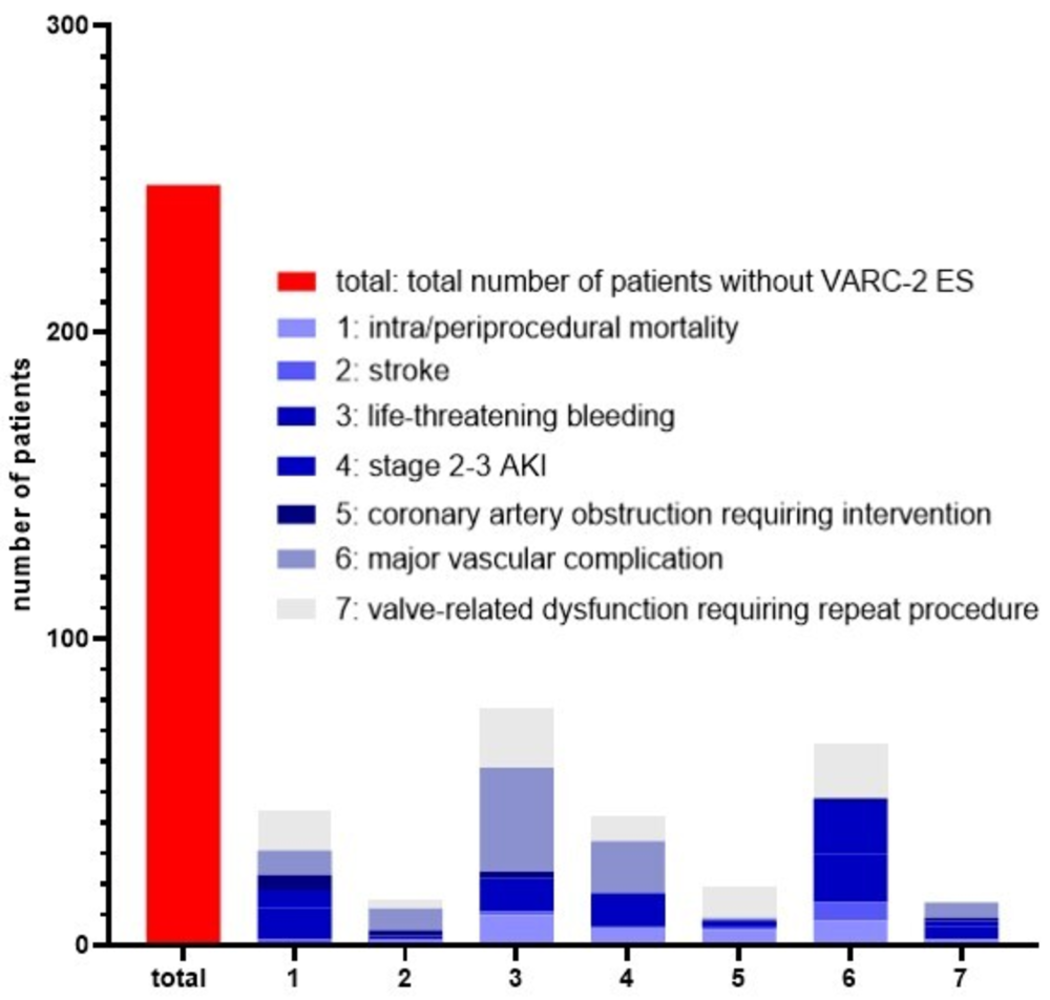

Supplement: Supplementary file 1 [file jcdd-10-00244-s001.zip › SupplementaryFigure1a.tiff]

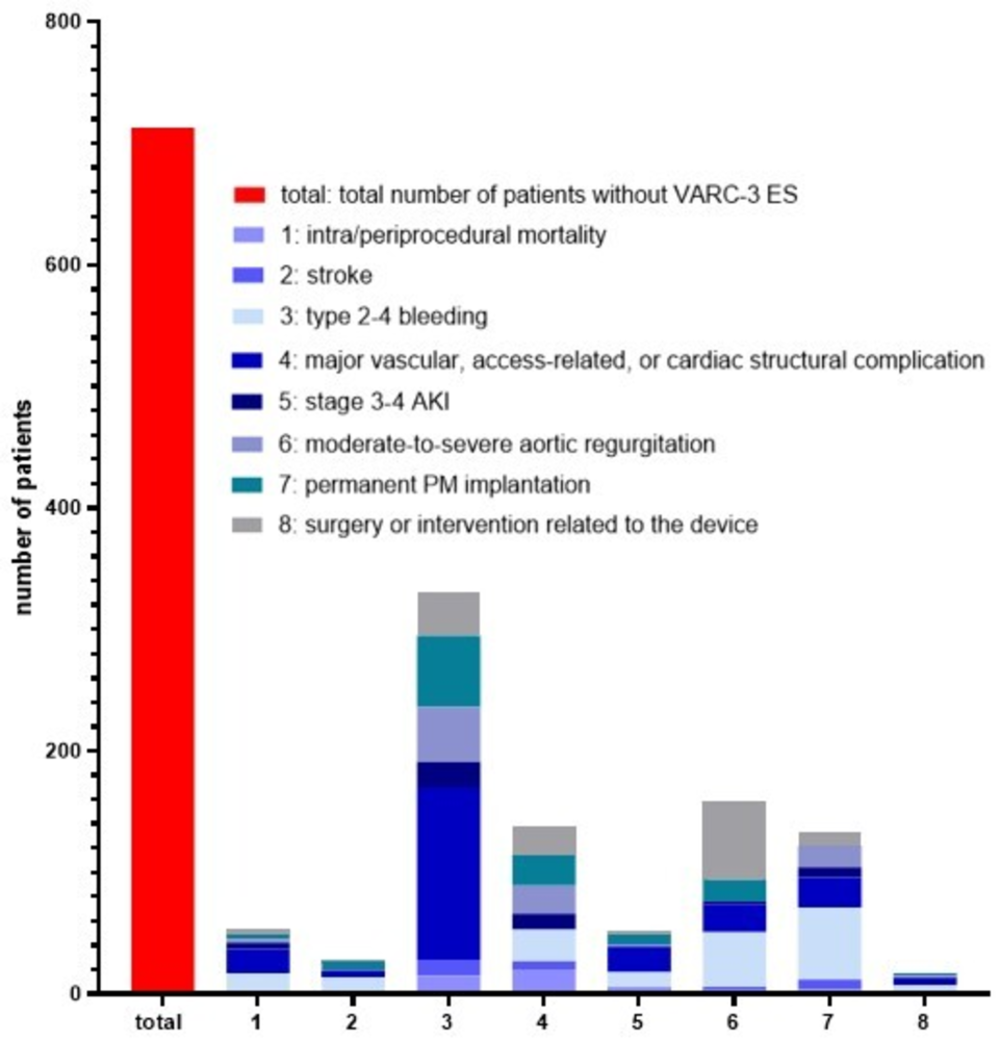

Supplement: Supplementary file 1 [file jcdd-10-00244-s001.zip › SupplementaryFigure1b.tiff]
